# Graphene-Iodine Nanocomposites: Highly Potent Bacterial Inhibitors that are Bio-compatible with Human Cells

**DOI:** 10.1038/srep20015

**Published:** 2016-02-04

**Authors:** Surajit Some, Ji Soo Sohn, Junmoo Kim, Su-Hyun Lee, Su Chan Lee, Jungpyo Lee, Iman Shackery, Sang Kyum Kim, So Hyun Kim, Nakwon Choi, Il-Joo Cho, Hyo-Il Jung, Shinill Kang, Seong Chan Jun

**Affiliations:** 1 School of Mechanical Engineering, Yonsei University, Seoul 120-749, South Korea; 2 Department of Dyestuff Technology, Institute of Chemical Technology, Matunga, Mumbai-400 019, India; 3 Department of Pathology, Severance Hospital, Yonsei University College of medicine, Seoul, Korea; 4 Center for BioMicrosystems, Brain Science Institute, Korea Institute of Science and Technology (KIST), Seoul, Korea

## Abstract

Graphene-composites, capable of inhibiting bacterial growth which is also bio-compatible with human cells have been highly sought after. Here we report for the first time the preparation of new graphene-iodine nano-composites via electrostatic interactions between positively charged graphene derivatives and triiodide anions. The resulting composites were characterized by X-ray photoemission spectroscopy, UV-spectroscopy, Raman spectroscopy and Scanning electron microscopy. The antibacterial potential of these graphene-iodine composites against Klebsiella pneumonia, Pseudomonas aeruginosa, Proteus mirobilis, Staphylococcus aureus, and E. coli was investigated. In addition, the cytotoxicity of the nanocomposite with human cells [human white blood cells (WBC), HeLa, MDA-MB-231, Fibroblast (primary human keratinocyte) and Keratinocyte (immortalized fibroblast)], was assessed. DGO (Double-oxidizes graphene oxide) was prepared by the additional oxidation of GO (graphene oxide). This generates more oxygen containing functional groups that can readily trap more H^+^, thus generating a positively charged surface area under highly acidic conditions. This step allowed bonding with a greater number of anionic triiodides and generated the most potent antibacterial agent among graphene-iodine and as-made povidone-iodine (PVP-I) composites also exhibited nontoxic to human cells culture. Thus, these nano-composites can be used to inhibit the growth of various bacterial species. Importantly, they are also very low-cytotoxic to human cells culture.

Graphene is a single-atom-thick sheet of dense honeycomb-arrayed structures composed of sp^2^-bondedcarbon atoms that has attracted enormous amount of interest due to its unique physical properties^1^. This material can be used in many fields including the biomedical field^2^ as biosensors^3^, biochips^4^, diagnostic devices^5^, implantable medical devices (e.g., prostheses)^6^, drug delivery systems^7^, and imaging probes^8^. Both graphene oxide (GO) and double oxidized GO (DGO) contain a wide range of reactive oxygen functional groups. These functional groups facilitate their use in bioengineering. Graphene derivatives exhibit a low production cost, large surface area, good colloidal behavior, and low cytotoxicity. The solubility of graphene derivatives in solvents, especially water, is highly important for bioengineering applications. The maximum solubility of these graphene derivatives insolvent depends on both solvent polarity and the extent of oxygen group surface functionalization. Previous reports have shown that graphene and its derivatives are biocompatible materials that allow the growth of human cells, with very limited or non-cytotoxicity^9^. These unique characteristics of graphene have motivated research groups to use these materials in tissue engineering, wound therapy, tissue implants, and drug delivery applications.

Several researchers have reported that graphene derivatives promote the adhesion and proliferation of different type of human cells^10^,^11^,^12^. One report has revealed that contact with GO reduced the growth of E. coli and Staphylococcus aureus bacterial cells about 51 and 61%, respectively. Similar results were been reported when these two microorganisms were exposed to GO nanowalls^13^. Moreover, graphene oxide film has been reported to produce inhibition zones in E. coli and S. aureus^14^. However, another report by Das *et al*. found that GO was not cytotoxic and lacked any antibacterial effect^15^. In summary, the numerous conflicting reports about the antimicrobial properties of GO.

Ma *et al*.^16^ reported that silver-modified graphene oxide nanosheets exhibited antibacterial activity against E. coli. Also, Bao *et al*.^14^ reported the preparation of a paper-like Ag nanoparticle/GO composite material via the *in-situ* reduction of absorbed Ag+ by hydroquinone (HQ) in a citrate buffer solution. Using a different approach, Liu *et al*.^17^ reported that Ag nanoparticles anchored on GO composites through a two-phase (toluene-water) process. In addition, Tang *et al*.^18^ reported that silver nanoparticles anchored on GO exhibited antibacterial properties against both the Gram-negative bacteria E. coli and the Gram-positive bacteria Staphylococcus aureus (S. aureus).

Finally, Ruiz *et al*. demonstrated that GO does not have inherent antibacterial, bacteriostatic, or cytotoxic effects in either bacteria ormammaliancells^19^. According to them, silver-doped GO films were able to produce inhibition zones and induce bacterial cell death; however silver is highly cytotoxic for cell cultures^20^.

In addition, graphene-PLL composite materials were reported as showing antibacterial effects against E. coli bacteria and are biocompatible with human cell cultures^21^. The antimicrobial activity of PLL against E. coli is well established^22^. Lee *et al*. were able to attach a greater number of PLL molecules to the graphene surface due its large surface area with oxygen functional groups, resulting in a product more potent than PLL alone^21^.

The iodine complex, which is formed by mixing polyvinylpyrrolidone (PVP) and iodine has been established as a way to take advantage of the desirable biocidal properties of iodine, while simultaneously avoiding its irritating side effects^23^. Hence, PVP-I has become the most usable attractive product, especially in surgery where it is used to coat the skin to prevent infection. Although povidone-iodine is claimed by many to be nonsensitizing and nonirritating to human tissue, cases of contact irritation and contact allergic dermatitis to PVP-I formulations have also been reported^24^. PVP is a water soluble polymer, and likewise, the as-made PVP-I complex is also water soluble. However, water solubility is undesirable for most of the applications of these formulations. Hence, there is a need for more effective and stable composites.

## Results

The main objective of this study was to prepare a series of graphene-iodine composites, based on the commercially available povidone-iodine (PVP-I) complex, with potent antibacterial activity. Both GO and DGO are known to contain many oxygen functional groups (e.g., hydroxyl, carboxyl, and epoxy groups), which facilitate the trapping of H^+^ ions. This action results in a net positive charge under highly acidic conditions (pH = 1)^25^. Accordingly, positively charged GO and DGO layers could be formed. The total amount of positive charges could be increase with increasing H^+^ ion concentration, assuming that many triiodide anion molecules are efficiently attached onto the positively charged GO and DGO surfaces. The surface area of these composites are very large compared with that of PVP-I alone; thus, GO-I and DGO-I composites exhibit improved performance compared with PVP-I alone. The improved performance is presumably a result of the large triiodide-containing surface area, which enables the nano-materials to contact multiple bacteria at once. The antibacterial reactivity of graphene-iodine composites would be higher than PVP-I alone; the mechanism of reactivity of those composites will be very similar to PVP-I^23^,^26^. However, the exact mechanism of the release of elemental iodine from the PVP-I molecule is not established^24^. According to previous reports, after iodine is released, it is toxic to microorganisms because it can combines irreversibly with tyrosine residues of proteins, interferes with the formation of hydrogen bonds by some amino acids and nucleic acids, oxidizes sulfhydryl groups, and reacts with sites of unsaturation in lipids^24^. We assumed that the antibacterial property of the graphene-iodine composites was dominated by the diffusion of iodine from those composites^26^. The growth of bacteria (Klebsiella pneumonia, Pseudomonas aeruginosa, Proteus mirobilis, Staphylococcus aureus, and E. coli) in the presence of graphene derivatives and their iodine composites was determined by evaluating antimicrobial activity. The biocompatibility of those materials were evaluated in the presence of human cells [WBCs, HeLa, MDA-MB-231, Fibroblast (primary human keratinocyte) and Keratinocyte (immortalized fibroblast)]. One previous report suggested that the SWCNT and PVP-I composite showed antibacterial activity against E.coli bacteria^27^. Presumably novel GO-I and DGO-I composites exhibit improved potent antibacterial activity with a very low-cytotoxic effect on human cells, whereas reported GO-Ag has high cytotoxic effect towards bacteria as well as human cells. However, till now no report has demonstrated that graphene-iodine composites have intrinsic antibacterial properties and are very low-cytotoxic to human cells.

### Characterization of Graphene-I Composites

**Figure 1:**
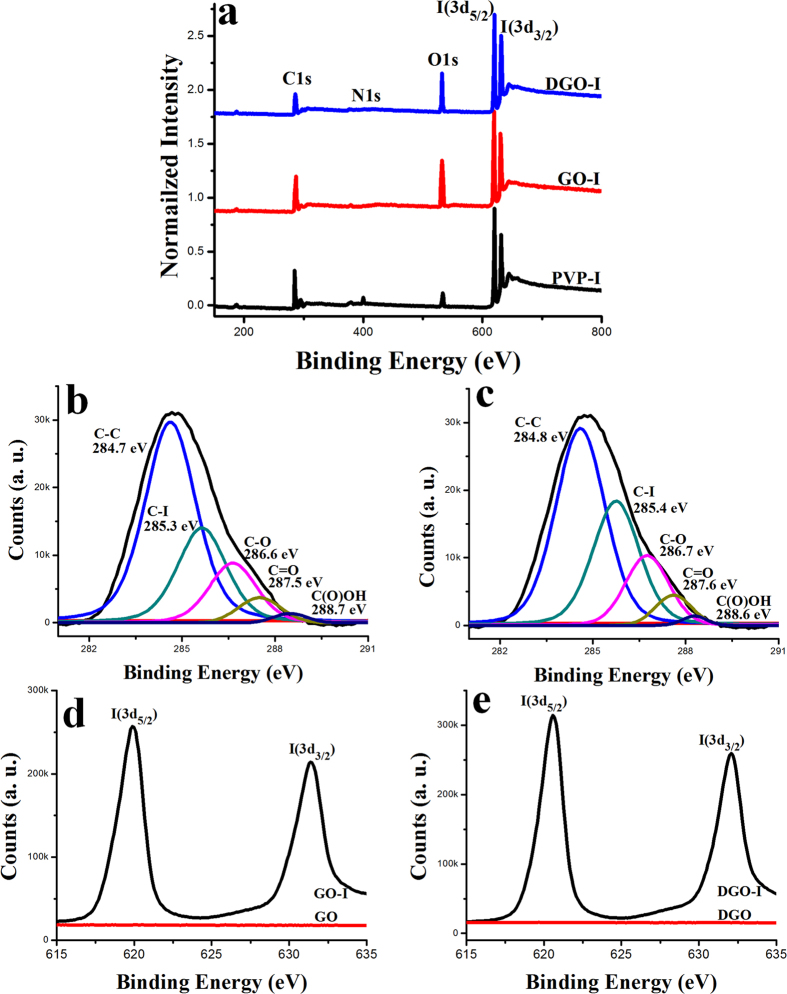
I (**a**) XPS survey spectra of DGO-I, GO-I, and PVP-I. C1s spectra of GO-I (**b**) and DGO-I (**c**). High-resolution I 3d spectra of GO-I (**d**) and DGO-I (**e**).

X-ray photoemission spectroscopy (XPS) was used to investigate the elemental composition and amount of materials. As-made GO sheets were synthesized from graphite powder using a modified Hummer’s method (Bay carbon, SP-2) and were purified as previously reported^28^. As-made DGO sheets were synthesized by a previously reported procedure^29^. Based on XPS analysis, the as-made GO had a low C/O ratio (~2) (Figure S2). The resulting DGO exhibited a low C/O ratio of ~1.2, corresponding to an increase in the oxygen content of the DGO nanosheets, indicating that the DGO contained the highest amount of oxygen-containing functional groups. The as-made GO-I and DGO-I composites were analyzed by XPS. Figure 1a shows the survey spectra of the GO-I, DGO-I and PVP-I; atomic iodine is detected in the form of triiodide and pentaiodide. The high-resolution C1s XPS spectrum of the as-made GO-I sheets showed a sharp peak at 284.7 eV, which corresponds to C-C bonds of carbon atoms in a conjugated honey-comb lattice. Peaks at 286.6, 287.5 and 288.7 eV could be assigned to different C-O bonding configurations (Fig. 1b)^28^. In comparison to the carbon XPS spectra after the doping, we can see the peak appeared at 285.3 eV after the doping that corresponds to the energy of the C–I bonds^30^,^31^. Similarly, the C1s XPS spectrum of the DGO-I sheets showed a sharp peak at 284.8 eV that also corresponds to C-C bonds of carbon atoms. Peaks at 286.7, 287.6 and 288.6 eV could be assigned to different C-O bonding configurations of the sp^2^ atomic structure of graphite (Fig. 1c)^28^. We also observed a peak appearing at 285.4 eV, after the iodine doping in DGO, which corresponds to the energy of the C–I bonds^30^,^31^. The C1s peak is observed at 284.7 and 284.8 eV for GO-I and DGO-I, respectively, which is a small shift from 284.6 eV (C-C bond in GO and DGO). Figure 1d,e show XPS core-level spectra of I 3d of GO-I and DGO-I, respectively. In both cases, two split peaks at 619.4 and 631.93 eV were observed, correspond to I 3d_3/2_ and I3d_5/2_, respectively, which are attributed to the atomic iodine in the GO-I and DGO-I composites^30^,^31^. As reported, the I 3d peak is different than that of pure iodine molecules (I_2_) as observed at a binding energy of 619.9 eV. Thus, I 3d XPS analysis assumes that the iodine atoms are well attached to the graphene surface and not pure iodine molecules, indicating an electrostatic interaction between iodine and oxygen functional groups. The doped iodine can form a chemical bond with the oxygen functional groups in the GO and DGO structures that involves electrostatic interaction. The quantitative analysis of the XPS spectra shows the presence of 12.2% iodine in as-made PVP-I, whereas GO-I and DGO-I have 15.3% and 19.1%, respectively. Thus, we concluded that the as made GO-I and DGO-I contain higher amounts of iodine in comparison to as made PVP-I. The I 3d XPS spectra of PVP-I are given in Figure S3.

**Figure 2:**
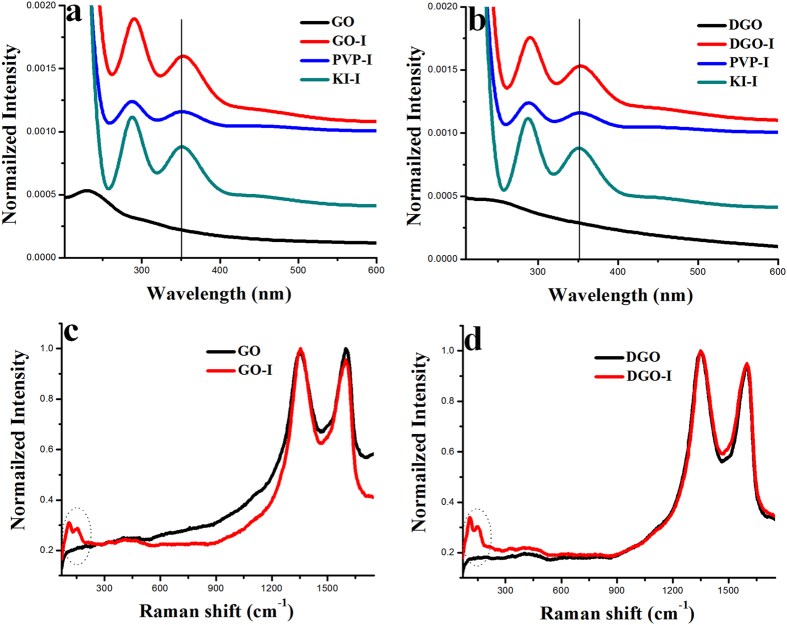
I (**a**) UV-visible spectroscopy of GO, GO-I, PVP-I, and KI-I. (**b**) UV-visible spectroscopy of DGO, DGO-I, PVP-I, and KI-I. (**c**) Raman spectra of GO and GO-I. (**d**) Raman spectra of DGO and DGO-I.

The presence of triiodide anion in the composites were tested by UV-spectra measurement. The main UV-vis absorption peak of GO and DGO appeared at 229.3^32^ and 253 nm, respectively (Fig. 2a,b). In the case of KI-I_2_ solution, the absorption peak appeared at 352.2 and a broad peak at 460 nm, in agreement with the reported λmax for the triiodide anion^33^. After the composite formation of triiodide anion with GO, DGO and PVP, we observed a small red shift relative to the triiodide anion peak. Specifically, the main absorption peaks of as made GO-I, DGO-I and PVP-I appeared in the UV-vis spectra at ~354 and ~465 nm, (~2–5 nm red shifts compared with the peak for KI-I_2_ solution alone), indicating the formation of composites with triiodide anion.

Raman spectroscopy provides a fast and easy structural and quality characterization of the as-made carbon materials. Raman spectra were collected from the as-made samples at an excitation wavelength of 514 nm, under ambient conditions, by dropping DI-water dispersions on a silicon (Si) substrate. Figure 2c,d show the Raman spectra of iodine-doped GO and DGO in comparison to only GO and DGO, respectively. The Raman spectra of as-prepared GO and DGO exhibited two remarkable peaks at about ~1353 and ~1600 cm^−1^, respectively, corresponding to the well-defined D and G bands (Fig. 2c,d). Generally, the G band is related to the E_2g_-vibration mode of sp^2^ carbon domains and can be used to explain the degree of graphitization. In contrast, the D band is associated with structural defects and partially disordered structures of the sp^2^ domains^28^. In our study, the I_D_/I_G_ ratio of the as-prepared DGO exhibited a significant increase in comparison to the as-made GO. In the case of GO, the I_D_/I_G_ ratio was 0.98 and, after double oxidation, the DGO sample was 1.02 due to two time strong oxidation procedure (Fig. 2c,d). The Raman spectroscopy in Fig. 2c,d shows that the D and G peaks of GO-I and DGO-I samples did not shift, compared to the GO and DGO samples. But, the I_D_/I_G_ ratio of GO-I and DGO-I exhibited a small increase in comparison to the as-made GO and DGO, respectively. At lower wave numbers, two new Raman peaks at 115 and 154 cm^−1^ were observed for GO-I and DGO-I compared with GO and DGO, which should be assigned to I_3_^−^ and I_5_^−^, respectively^30^,^31^. On the other hand, no Raman peak was observed corresponding to molecular iodine (I_2_) (181 cm^−1^), which further negates the possibility of physical accumulation of molecular iodine on the as-made material surface^31^.

**Figure 3:**
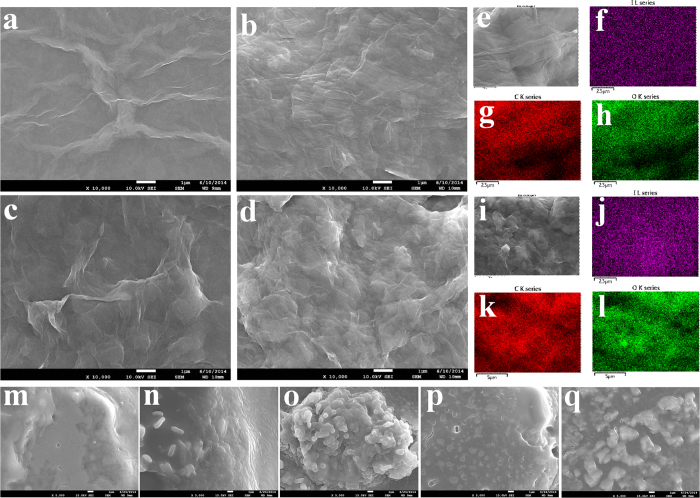
I SEM images of different materials. SEM images of (**a**) GO, (**b**) GO-I, (**c**) DGO, (**d**) DGO-I, (**e**) Element mapping of GO-I composite: (**f**) I atoms (**g**) C atoms (**h**) O atoms. (**i**) Element mapping of DGO-I composite: (**j**) I atoms (**k**) C atoms (**l**) O atoms. (**m**) Klebsiella pneumonia bacterial-biofilm-containing DGO after 12 h. (**n**) Pseudomonas aeruginosa bacterial-biofilm-containing DGO after 12 h. (**o**) Proteus mirobilis bacterial-biofilm-containing DGO after 12 h. (**p**) Staphylococcus aureus bacterial-biofilm-containing DGO after 12 h. (**q**) E.coli bacterial-biofilm-containing DGO after 12 h.

Scanning electron microscopy (SEM) analysis was used to observe the surface morphology of different materials (Fig. 3). We observed a thin and wrinkled GO sheets in the SEM images of GOs (Fig. 3a). The as-made GO-I showed different morphology in comparison to only GO (Fig. 3b). The SEM images of DGO revealed a more wrinkled morphology compared with that of GO (Fig. 3c). As-made DGO-I also showed different surface morphology in comparison to only DGO (Fig. 3d). We also measured the surface morphology of PVP-I and as made only KI-I_2_ complexes to compare with other composites (Figure S4). To demonstrate the distribution of iodine atoms on the GO-I and DGO-I surface, the element mapping in the selected area is shown in Fig. 3e–l. From the element mapping, we clearly observed the uniform distribution of C, O and I atoms in our composites.

The surface area of DGO-I and GO-I were measured ~165 m^2^g^−1^ and ~149 m^2^g^–1^ respectively, which were higher than that of the PVP-I (~12 m^2^g^−1^).

### Bacterial Proliferation in the Presence of GO, DGO and iodine Composites

**Figure 4:**
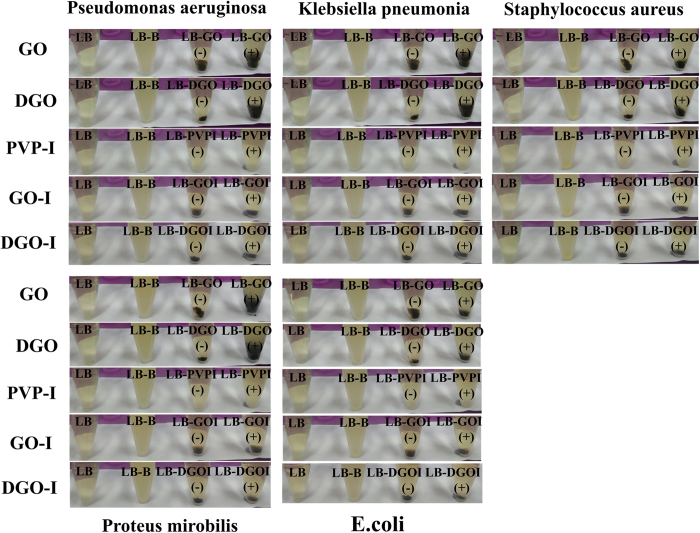
I Bacterial growth in four test tubes containing the following components after 9 h: LB, LB-bacteria, LB-composites, and LB-composites-bacteria. The materials used were GO, DGO, PVP-I, GO-I, and DGO-I with bacteria Pseudomonas aeruginosa, Klebsiella pneumonia, Staphylococcus aureus, Proteus mirobilis, and E.coli.

To determine the antibacterial effect of GO, DGO, GO-I and DGO-I composites, samples containing 5 mL of Luria-Bertani (LB) nutrient broth in 20 mL test tubes were incubated with prepared materials to a final concentration of 20 μg/mL. As-made materials were then inoculated with E. coli, Klebsiella pneumonia, Proteus mirabilis, Pseudomonas aeruginosa, and Stapylococcus aureus bacterial cells to a 0.01 optical density (OD)^19^. Antibacterial experiments were performed with the same amount of dose of materials 1, 2, 5, and 6 (Figure S1), including PVP-I. The experimental controls were prepared by inoculating five kinds of bacteria to an OD of 0.03 in 5 mL of LB broth, which means without any of the composites. We examined the supernatant of the cultures at 3 hour intervals to appraise the bacterial growth, without disturbing the precipitate at the bottom of each test tubes. At 3, 6, 9, and 12 h, we examined the bacterial growth by measuring the absorbance of supernatant of the cultures at 600 nm. The ODs of iodine composites (materials 5 and 6) only did not increase after 3 h, whereas the ODs of PVP-I sample increased very little. Whereas the other materials (without iodine composites) significantly increased compared with the control samples (i.e., without bacteria). In samples containing PVP-I, the ODs increased after 6 h compared with GO-I and DGO-I samples. However, the ODs were still very low in comparison to containing only bacteria samples. In the case of all bacteria, culture tubes containing GO-I (material 5), and DGO-I (material 6) did not have any discriminable difference in ODs in comparison to control samples (Fig. 4). The ODs of PVP-I-containing culture tubes increased in comparison to GO-I (material 5), and DGO-I (material 6). Surprisingly, samples containing GO-I (material 5), and DGO-I (material 6) did not show any discriminable difference in ODs (average absorbance in the range of 0.00–0.04) after 12 h compared with the control samples. The ODs of GO and DGO (without iodine) containing samples gradually increased with time and became saturated, in comparison to control samples. After ~9 h, samples containing only bacteria appeared to be further turbid and had the highest amount of ODs, which may be represent a saturation point compared with control samples (except samples 5 and 6).

**Figure 5:**
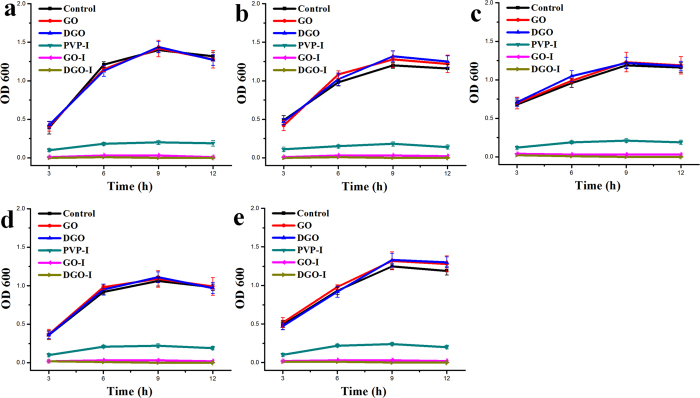
I Graphs showing bacterial growth levels in the supernatant of samples containing prepared composites: (**a**) E. coli, (**b**) Klebsiella pneumonia, (**c**) Proteus mirobilis, (**d**) Pseudomonas aeruginosa, and (**d**) Staphylococcus aureus.

The plot of ODs shown in Fig. 5 demonstrates that the antibacterial property of GO-I and DGO-I composites was higher than that of PVP-I alone. Previous studies have already shown that when a dispersed GO solution is added to a media solution, which containing salts, GO aggregates and precipitates out of the suspension due to producing low-densityaggregates^19^,^34^. The presence of a large amount of cells in biofilms has also been reported, indicating that GO has no antibacterial effect when added to liquid media^19^. After 12 h, bacterial grown experimental DGO and DGO-I samples were analyzed by SEM (Fig. 3, S5). According to the SEM images, the precipitated samples of DGO were covered by a thick bacterial biofilm (Fig. 3m–q) containing a large mass of aggregated cells in comparison to the control sample (LB broth and DGO, Fig. 3c), whereas bacterial experiment with DGO-I did not show any biofilm formation (Figure S5). According to the experimental results, the antibacterial property of DGO-I composites was enough to prevent bacterial growth in LB broth, in comparison to that of PVP-I composites. Thus, GO-I and DGO-I composites could act as strong antibacterial agents. The ODs shown in Fig. 5 represent the antibacterial activity of various iodine composites. The DGO-I composite was the most effective inhibitor of bacterial growth and was more potent than the GO-I composite, whereas the GO-I and DGO-I composites were both more potent inhibitors than PVP-I. Slowly liberated free iodine, from the iodine complexes in solution, kills eukaryotic or prokaryotic cells through iodination of lipids and oxidation of cytoplasmic and membrane compounds^26^. Because triiodide has an anionic charge, it attaches to the positively charged oxygen groups of the large surface areas of GO and DGO, via electrostatic interactions. Thus, more cationic oxygen functional groups of graphene nanomaterial with a large surface area can conduct the more attachment of anionic triiodide, increasing antibacterial property in comparison to that of PVP-I, which does not have a high surface area. We hypothesized that the amount of triiodide anion attached to the GO surface should be less than the amount in comparison to that of DGO-I composite, resulting in lower antibacterial property. Indeed, according to our experimental results, DGO functionalized with more oxygen functional groups which enabled the more triiodide-attached composites, thus producing an improved antibacterial agent (Fig. 5).

To further evaluate the antibacterial effect of graphene derivative-I composites, we performed a live/dead assay with the DGO-I composite. All bacteria were cultured in 1 mL of Luria-Bertani (LB) nutrient broth in 5 mL test tubes with OD of 0.1 (~4 × 10^7^ cells). Then all the bacterial cells were incubated for 1 h in presence of DGO-I composite with a final concentration of 20 μg/mL. According to previous reports, SYTO 9 and propidiumiodide (PI) dye were used to perform the live/dead assay experiment^21^. As only living and dead bacteria will be stained by SYTO 9 or PI, bacteria can easily be dappled and confined between abiotic particles. The positively charged fluorescent label PI can stain only the dead bacterial cells as it unable to pass through the positively charged cell membranes of the living cells. In contrast to the fluorescent label PI molecules, the neutral SYTO 9 molecules can traverse the cell membrane of both the living and dead cells. However, when dead cells are labelled with a mixture of SYTO 9 and PI, the green fluorescence caused by SYTO 9 will be subdued by a fluorescence resonance energy transfer (FRET) effect, causing the dead cells to fluoresce in red. It has been reported that the combination of SYTO 9 and PI is well suited stain system for eukaryotic cells^35^. In Figure S6 are presented the staining results for all analyzed species. The SYTO 9 and PI overlay images in Figure S6 (1a–5a) clearly show the presence of only live cells in control treated samples and Figure S6 (1b–5b) show the presence of only dead cells with composites treated samples. The relative average intensities of only dead cells for each condition were calculated to be 3–5% and 95–97% for the control and the DGO-I-treated samples, respectively [Figure S6 (1c–5c)]. The experimental data were quantified by taking the ratio of the number of dead cells to the total number of cells for both the conditions, the control and the DGO-I treated samples (n = 3). The differences between the percentages for the control and the as-made material-treated samples were found to be statistically remarkable using a t test (p < 0.005). Accordingly to the live/dead assay experiment, the DGO-I composite had high antibacterial activity on all five kinds of bacterial cells.

**Figure 6:**
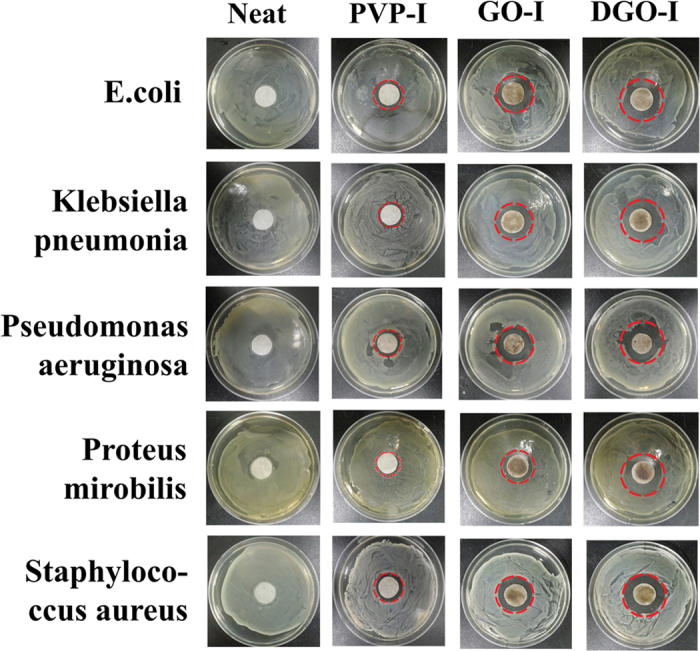
I Photographs of the inhibition zone by the disk diffusion assay of E. coli, Klebsiella pneumonia, Pseudomonas aeruginosa, Proteus mirobilis, and Staphylococcus aureus bacteria with neat, PVP-I, GO-I, and DGO-I coated filter paper.

The antibacterial activities of the GO-I and DGO-I composites were also investigated qualitatively by the disk diffusion assay (Fig. 6). First, we coated sterile filter papers with neat, GO, DGO, GO-I, DGO-I, and PVP-I materials. Filter papers were allowed to dry and were then incubated for 24 h at 37 °C on LB culture plates containing each bacterium at a concentration of 1 × 10^9^ cells/mL. Filter paper without any material coating (neat) served as a control sample. Bacteria growth was observed with the naked eye in the cases of GO- and DGO-coated, and neat filter paper samples with large bacteria colonies (Figure S7). We did not find an inhibition zone in either the GO- and DGO-treated samples (Figure S7), or the control sample (Fig. 6), since GO and DGO have no antibacterial activity. However, after coating the filter paper with GO-I, DGO-I, and PVP-I composites, the inhibition zones were obviously visible. The average zones of inhibition of bacterial growth, as measured in millimetres from the edge of the filter paper to the point of uninhibited bacterial growth, were as summarized in Table 1. The diameter of the inhibition zone of each bacteria treated with DGO-I is a little bit larger than that treated with GO-I, and those are even larger than PVP-I, under the same conditions (Table 1). The inhibition zones are generated due to the diffusion of iodine from the graphene-iodine complexes to the agar, rather than to the release of iodine vapor above the plate. The differences in the inhibition zones represent the different sensitivities of the organisms to the iodine and also to the different amounts of iodine that diffuse from the various iodine composites. The iodine is slowly released from these as-made complexes (Table S1)^27^.

### Cytotoxicity of GO, DGO and iodine Composites on Human Cells

**Figure 7:**
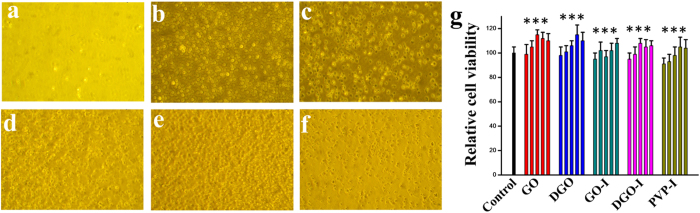
I Growth of Human white blood cells on (**a**) glass coverslips, (**b**) GO, (**c**) DGO, (**d**) GO-I, (**e**) DGO-I, and (**f**) PVP-I (20×). (**g**) *In vitro* concentration-dependent (100, 50, 25, 12.5, and 6.125 μg/mL, respectively from left to right) cell viability of WBCs. The cells were incubated with free GO, DGO, GO-I, DGO-I, and PVP-I for 24 h as indicated. Data represent mean ± Standard Error of the Mean (SEM)(n = 6)(***p < 0.05 versus PBS, one-way ANOVA).

We evaluated the effect of composite (materials 1, 2, 5, 6, and PVP-I) films on human cell attachment and growth using common human WBCs, HeLa and MDA-MB-231cell lines^22^,^36^,^37^. Various composites were coated on glass coverslips by spin-casting samples with 100 μL of a 1 mg/mL solution^21^. Only glass coverslips and GO/DGO composite-coated coverslips were placed in a culture dish followed by the addition of the cell culture media and different human cells. Cells were allowed to attach and grow on the coverslips. Those cells attachment was evaluated by light microscopy at different times. Figure 7 and S8 are shown the representative images of different cell morphologies of WBCs, HeLa and MDA-MB-231cells after incubation for 24 h. According to the experimental results, the human cells efficiently attached to and grew on glass coverslips coated with different composites (Fig. 7b–f). The micrographs of WBCs showed marked morphological changes and spreading on coverslips coated with composites in comparison to the control glass coverslips (Fig. 7a), which is well known characteristic of effective cell attachment and cell growth (Fig. 7). In contrast the control experiments with only glass coverslip, few cells were attached, indicating a lack of enlargement and growth of WBC cells (Fig. 7a). These data suggest that the graphene-iodine composites (Fig. 7d,e) also allow more efficient growth of human cells in comparison to the as-made PVP-I, which implies that these materials are not cytotoxic to human cell culture with a used amount of material. Similar findings were observed for HeLa and MDA-MB-231 cells (Figure S8). Taken together, these results indicate that all of the prepared iodine composites are suitable for human cell attachment and growth.

**Figure 8:**
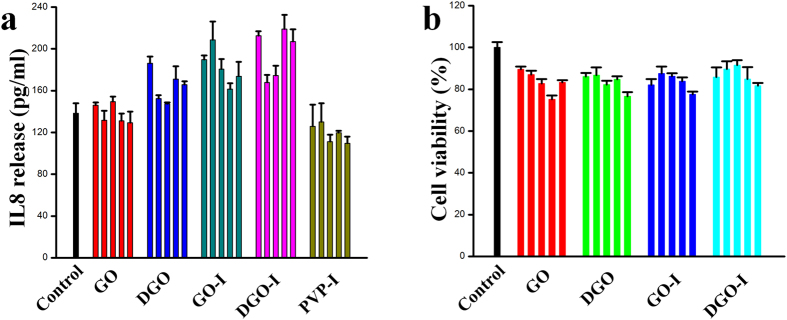
I The IL-8 release (ELISA) (**a**) and the cytotoxicity test (EZ-CYTOX assay) (**b**) for as-made GO, DGO, GO-I, DGO-I, and PVP-I with different concentration (6.125, 12.5, 25, 50, and 100 μg/mL, respectively from left to right) for human skin cells [Fibroblast (primary human keratinocyte), Keratinocyte (immortalized fibroblast)].

To evaluate and compare the *in vitro* cytotoxicity of the composites, we analysed the effects of GO, DGO, GO-I, DGO-I, and PVP-I on the cell viability of common human WBCs, HeLa and MDA-MB-231cell lines. As expected, cell viability were greater than ~98% for GO and DGO was as high as 100 μg mL^−1^ for all cell lines, indicating that the graphene derivatives were biocompatible (Fig. 7g, S8)^38^. As depicted in Figure 7g and S8, no noticeable amount of cells were observed by as-made GO-I, DGO-I at the high concentration of 100 μg mL^−1^. Moreover, PVP-I also exhibited a biocompatibility at the same condition. Therefore, we can conclude that our as made GO-I and DGO-I derivatives are non-cytotoxic to human cells. We have also analyzed the cytotoxicity test (EZ-CYTOX assay) and the IL-8 release (ELISA) for our as-made nanomaterials for human skin cells [Fibroblast (primary human keratinocyte), Keratinocyte (immortalized fibroblast)] (Fig. 8). Actually, EZ-cytox assay was also performed with PVP-I treated cultures. However, the absorbance from the PVP-I treated cultures supernatants did not reflect cell viabilities because PVP-I still remained in the supernatants even after centrifugation at 12,000 g. In case of EZ-CYTOX we have observed that our as-made nanomaterials are very low toxic.

## Discussion

This was a comprehensive study of antibacterial effects of triiodide molecule onto the graphene (GO and DGO) surface based on their number of oxygen containing functional groups. GO-I, DGO-I composites were prepared by the interactions between iodine and the number of oxygen containing functional groups of respective graphene materials. The antibacterial potency increases as the number of oxygen containing functional groups on graphene derivatives increase. This increase is due to the resultant large surface area, which can undergo extensive iodine interactions via electrostatic interaction, thereby generating graphene-iodine composites (GO-I and DGO-I). Therefore, this is the first report to show graphene-iodine composites can be highly effective antibacterial agents with very low-cytotoxic effects for human cell cultures. The most effective part is that DGO-I composites, featured the highest antibacterial activity ever reported with biocompatible for human cells cultures. Our graphene and iodine composites are easy to fabricate, contained higher amounts of iodine, and exhibited superior antibacterial activity compared with to as-made PVP-I at the same doses.

In summary, this study demonstrates that graphene alone has no antibacterial properties, which is consistent with previous reports. We synthesized novel graphene-iodine composites via the electrostatic interactions between triiodide anions and GO and DGO derivatives synthesized to have positively charged oxygen functional groups. The triiodide generated a larger iodine source on DGO-I composites. As a result, DGO-I composites exhibited the most potent antibacterial activity compared to the other compounds in the study. Applications of iodine composites were explored by fabricating antibacterial materials that can be act as a bacterial growth inhibitor. In addition, GO-I and DGO-I composites were shown to be biocompatible for different human cell cultures. This is the first report demonstrating that graphene-iodine composites can provide potent bacterial growth inhibitor activity with very low-cytotoxic to human cells. The results of this study thus provide insight into the biological properties of iodine nano-composites and indicate their potential biomedical and biotechnological applications.

## Methods

### Preparation of Graphene Oxides (GOs)

GO sheets were prepared from natural graphite powder using the modified Hummer’s method with sulfuric acid, potassium permanganate, and sodium nitrate^28^.

### Synthesis of Double Oxidized Graphene Oxide (DGO)

DGO sheets were synthesized by the reported procedure from our prepared GO^29^.

### Synthesis of PVP-I Composite

200 mg of KI and 100 mg of I_2_ were mixed with 6 mL of DI-water and sonicated for 30 min, followed by the addition of 185 mg of PVP. The resulting complex was centrifuged at 14,000 rpm for 20 min, washed three times with benzene, and the resulting pellet was dried under vacuum^26^.

### Synthesis of GO-I, and DGO-I Composites

GO and DGO nanosheets were dispersed in deionized distilled water (~10 mg/mL), then the solution was adjusted to ~pH 1, and finally mixed with as-made KI-I_2_ solution and sonicated for 1h. The resultant solutions were stirred at ~60 °C for 1 h followed by centrifugation at 14,000 rpm for 20 min, washed three times with benzene, and the resulting pellet was dried under vacuum.

### Bacterial Cell Culture

Klebsiella pneumonia (strain MRKP), Pseudomonas aeruginosa (strain PA14), Proteus mirobilis (strain PR03), Staphylococcus aureus (strain RN4220), and Escherichia coli (strain DH5R), were grown alone, and with different composites, in Luria-Bertani (LB) broth in 20 mL test tubes and incubated for 12 h at 37 °C to a final concentration of 20 μg/mL^19^.

### Bacterial Growth on a Solid Surface

The sterile filter papers were dipped into GO, DGO, GO-I, DGO-I, and PVP-I solution (1 mg/mL) and allowed to dry in a vacuum. The resulting filter papers were then placed on a sterile LB culture plate coated with bacteria solution containing 1 × 10^9^ cells/mL, and incubated for 24 h at 37 °C^19^.

### Live/dead assay

Bacteria samples containing 1 mL of Luria-Bertani (LB) nutrient broth in 5 mL test tubes were incubated for 1 h with DGO-I composites to a final concentration of 20 μg/mL. The initial OD of the bacterial solution was 0.1 (4 × 10^7^ cells). After that, a Live/Dead Bac-Light Bacterial Viability Kit (L-7007, Molecular Probes) was used to label bacterial cells. The staining procedure was followed according to the protocol provided by Molecular Probes (MP07007). Live bacteria fluoresce green (SYTO 9) and dead bacteria fluoresce red (propidium iodide)^21^. WBC cell line (jurkat cell) and breast cancer cell lines (HeLa Cell, MDA-MB-231 Cell) were cultured on plate at 37 °C under 5% CO_2_ with various concentrations (from 6.125, 50, 100, 500, and 1000 μg/mL) of 1, 2, 3, 4, 5, 6 and 7 by diluted with DI. The viability of cells on the seven materials was estimated after 24 h. Following incubation, the adherent cells were detached from the culture plate with trypsin, and counted. Cell death was analyzed by trypan blue dye (Sigma Aldrich, Germany) on cell lines. Equal amounts of cell suspension and trypan blue were mixed and this was analyzed under a microscope. The cells which were viable excluded the dye and were colorless while those whose cell membrane was destroyed were blue. Viable and dead cells were quantified by fluorescence microscopy. The concentrations of cells were measured using a hemocytometer^36^,^37^.

### Cell Culture

We used a human T-cell lymphoblast-like cell line (Jurkat cells). The cells were cultured in RPMI 1640 medium (Gibco) supplemented with 10% fetal bovine serum (FBS) and 1% penicillin and streptomycin (PS). These cells were incubated at 37 °C with 5% CO_2_. The medium was changed every two days. For experiments, 1.5 × 10^5^ cells were seeded in a culture dish containing the glass slides. After 24 h, the morphology of cell growth was observed under a microscope^39^. As common cancer cell lines, we used HeLa Cell Line human epitheloid cervix carcinoma (HeLa Cell Line) and human human breast adenocarcinoma (MDA-MB-231 Cell Line). The cells were cultured in RPMI 1640 medium (Gibco) supplemented with 10% fetal bovine serum (FBS) and 1% penicillin and streptomycin (PS). These cells were incubated at 37 °C with 5% CO_2_. The medium was changed every two days. In case of cancer cell lines, we detached from the plate by trypsin treatment because these two cell lines are adhensive cell line. For experiments, 1.5 × 10^5^ cells were seeded in a culture dish containing the glass slides. Adhensive cell lines were allowed to attach and develop on the slides. After 24 h, the morphology of cell growth was observed under a microscope^36^,^37^.

### Cytotoxicity Assay

WBCs were seeded into a 48-well cell-culture plate at 37 °C under 5% CO_2_ with various concentrations (6.125, 12.5, 25, 50, and 100 μg/mL) of solutions 1, 2, 5, 6 and PVP-I, diluted with DI. The viability of cells on the seven materials was estimated after 24 h. Cell death was analyzed by trypan blue dye (Sigma Aldrich, Germany) on the Jurkat cell line. Following incubation, equal amounts of cell suspension and trypan blue were mixed and this was analyzed under a microscope. The cells that were viable excluded the dye and were colorless, while those whose cell membranes were destroyed turned blue. Viable and dead cells were quantified by fluorescence microscopy. The concentrations of cells were measured using a hemocytometer. All experiments were performed under the same conditions, and the relative cell viability (%) was expressed as a percentage relative to the untreated control cells^40^.

### Cell culture

Primary human epidermal keratinocytes (Lonza, Basel, Switzerland) were cultured in KGM-Gold™ Keratinocyte Growth Medium supplemented with KGM-Gold SingleQuot Kit (Lonza, Basel, Switzerland). BJ-5ta human fibroblasts (ATCC, VA, USA) were cultured in DMEM (Lonza, Basel, Switzerland) supplemented with 10% heat inactivated fatal bovine serum (Corning, NY, USA) penicillin (100 U/ml) (GIBCO, MA, USA) and streptomycin (0.1 mg/ml) (GIBCO, MA, USA)^41^.

### Cytotoxicity test

Cytotoxicity of various graphene-iodine nanocomposites (GO, GO-1, DGO, DGO-1, PVP-1) were assessed on immortalized human fibroblasts using EZ-cytox assay (Daeillab service, Seoul, Korea). Fibroblasts at passage 9 were seeded into the wells of a 96-well plate (Costar, IL, USA) at a density of 1 × 10^4^ cells per well in 50 μL of phenol-red free DMEM culture medium. On the other hand, nano-material solutions at various concentrations (200, 100, 50, 25, and 12.5 μg/mL) were freshly prepared by diluting 1 mg/mL stock solutions of various nano-materials with the culture medium. To obtain well-dispersed nano-material suspensions, all the stock and the diluted solutions were sonicated for 30 min before using. After 12 h of seeding the cells, 50 μL of the nano-material solutions were added to each well in which their final concentrations were 100, 50, 25, 12.5, or 6.25 μg/mL. The cells were cultured with the nano-materials for 20 h and 10 μL EZ-cytox solution was added to each well. Then, the cells were incubated for a further 4 h to allow the color of the culture supernatants to be changed by reaction between viable cells and EZ-cytox solution. After the reaction, nano-material-free supernatants were obtained by spinning down nano-materials with 12,000 g. The nano-material-free supernatants were transferred to a fresh 96-well plate and absorbance was read at 450 nm to determine cell viability of each well. Finally, the cell viabilities of nano-material-treated cultures were expressed as percentages of nano-material-untreated control cultures^41^.

### Cytokine release assay_IL-8 ELISA

Human primary keratinocytes at passage 5 were seeded into the wells of a 24-well plate (Costar, IL, USA) at a density of 5 × 10^4^ cells per well in 500 μL of culture medium. On the other hand, nano-material solutions at various concentrations (200, 100, 50, 25, and 12.5 μg/mL) were freshly prepared by diluting 1 mg/mL stock solutions of various nano-materials with culture medium. To obtain well-dispersed nanomaterials suspension, all the stock and the diluted solutions were sonicated for 30 min before using. After 12 h of seeing the cells, 500 μL of the nano-material solutions were added to each well in which their final concentrations were 100, 50, 25, 12.5, or 6.25 μg/mL. After incubation of the cells the nano-materials for 24 h, the culture supernatants were collected to analyze amounts of IL-8 released from the cells using ELISA. Prior to ELISA test, nano-materials in supernatants were spun down at 12,000 g to reduce potential interference in antibody reaction by nanomaterials. The Assay was performed using a commercial —ELISA kit (Thermo Fisher scientific Pierce, IL, USA), following manufacturer’s instructions^41^,^42^,^43^.

## Additional Information

**How to cite this article**: Some, S. *et al*. Graphene-Iodine Nanocomposites: Highly Potent Bacterial Inhibitors that are Bio-compatible with Human Cells. *Sci. Rep*. **6**, 20015; doi: 10.1038/srep20015 (2016).

## Supplementary Material

Supplementary Information

## Figures and Tables

**Table 1:** I Inhibition zone on solid LB plate by the treatment with different composites.

**Bacteria Name**	Inhibitionzone withGO (mm)	Inhibitionzone withDGO (mm)	Inhibitionzone withPVP-I (mm)	Inhibitionzone withGO-I (mm)	Inhibitionzone withDGO-I (mm)
Klebsiella pneumoniae	–	–	19.1	25.8	35.1
Proteus mirabilis	–	–	18.2	23.2	32.6
Pseudomonas aeruginosa	–	–	21.4	28.8	35.9
Stapylococcus aureus	–	–	21.1	26.1	32.2
E. Coli	–	–	23.6	28.8	36.1
