# Supplementary material for: Graphene-Iodine Nanocomposites: Highly Potent Bacterial Inhibitors that are Bio-compatible with Human Cells
Source: Sci Rep. 2016 Feb 4;6:20015. doi: 10.1038/srep20015 (PMC4740772; doi:10.1038/srep20015)
Supplement: Supplementary Information [file srep20015-s1.doc]

Graphene-Iodine Nanocomposites: Highly Potent Bacterial Inhibitors that are Bio-compatible with Human Cells

**Surajit Some^1,2^*, Ji Soo Sohn^1^, Junmoo Kim^1^, Su-Hyun Lee^1^, Su Chan Lee^1^, Jungpyo Lee^1,4^, Iman Shackery^1^, Sang Kyum Kim^3^, So Hyun Kim^4^, Nakwon Choi^4^, Il-Joo cho^4^, Hyo-Il Jung^1^, Shinill Kang^1^, and Seong Chan Jun^1^***

^1^School of Mechanical Engineering, Yonsei University, Seoul 120-749, South Korea

^2^Department of Dyestuff Technology, Institute of Chemical Technology, Matunga, Mumbai-400 019, India
^3^Department of Pathology, Severance Hospital, Yonsei University College of medicine, Seoul, Korea

^4^Center for BioMicrosystems, Brain Science Institute, Korea Institute of Science and Technology (KIST), Seoul, Korea

E-mail: scj@yonsei.ac.kr (S.C. Jun), sr.some@ictmumbai.edu.in (S. Some)

**Materials**

Natural graphite (Bay Carbon, SP-1 graphite), sulphuric acid (95-97 %), hydrogen peroxide (30 wt. %), potassium permanganate, sodium nitrate, hydrochloric acid, polyvinylpyrrolidone, potassium iodide, iodine and benzene were obtained from commercial sources and used as received.

**Characterization**

All X-ray photoemission spectroscopy (XPS) measurements were made by a SIGMA PROBE (ThermoVG, U.K.) with a monochromatic Al-Kα X-ray source at 100 W. All UV-vis absorption spectra were recorded using a double-beam UV-1650PC spectrophotometer (Shimadzu). Raman spectroscopy measurements were performed by using a micro-Raman system (Renishaw, RM1000-In Via) with an excitation energy of 2.41 eV (λ = 514 nm). The microstructure was observed by field emission scanning electron microscopy (FE-SEM; JSM-6701F/INCA Energy, JEOL).


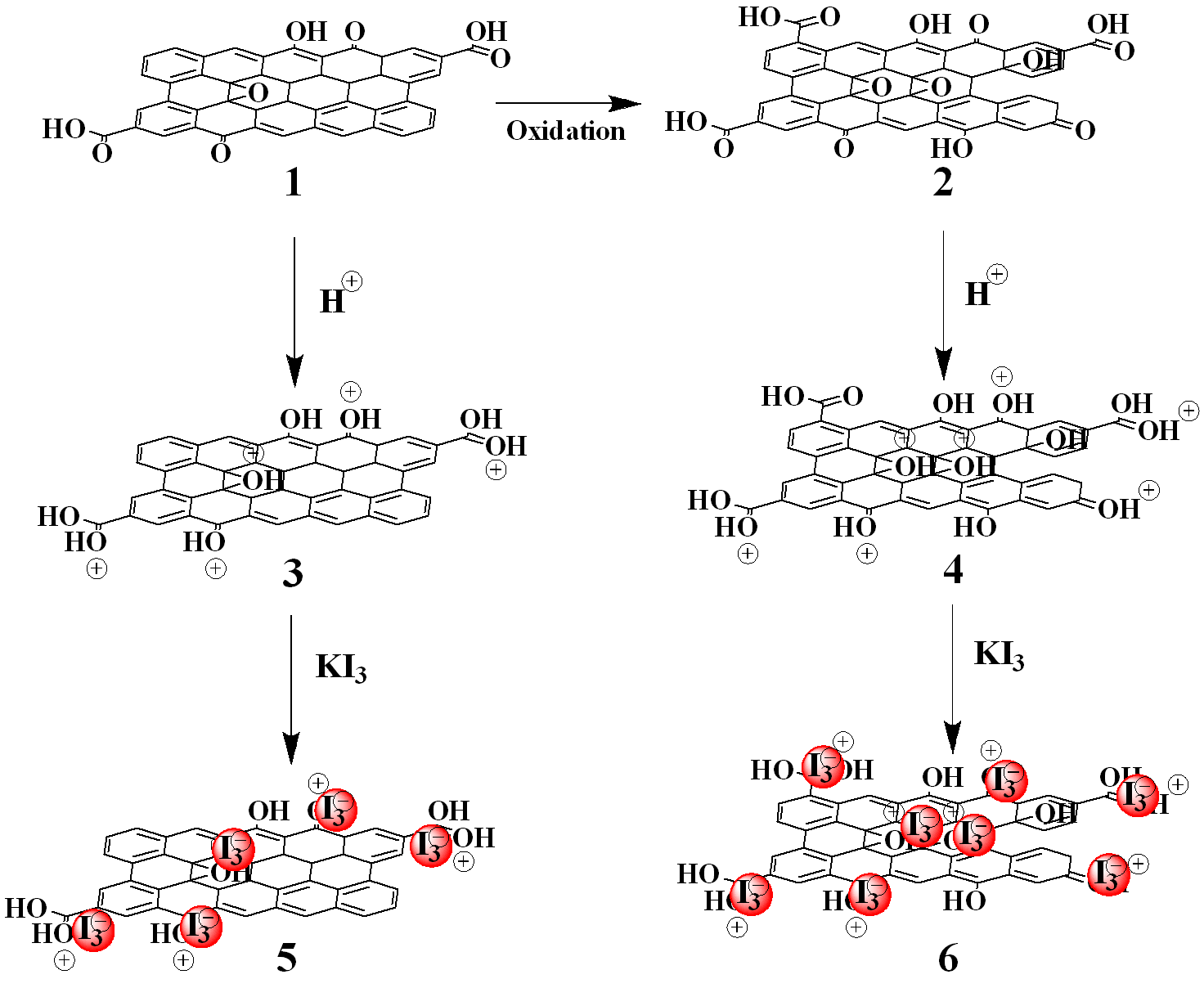


**Figure S1.** Schematic diagram of the preparation of various composites synthesized from graphene oxide. 1, GO; 2, DGO; 3, GO derivative; 4, DGO derivative, 5, GO-I; 6, DGO-I.


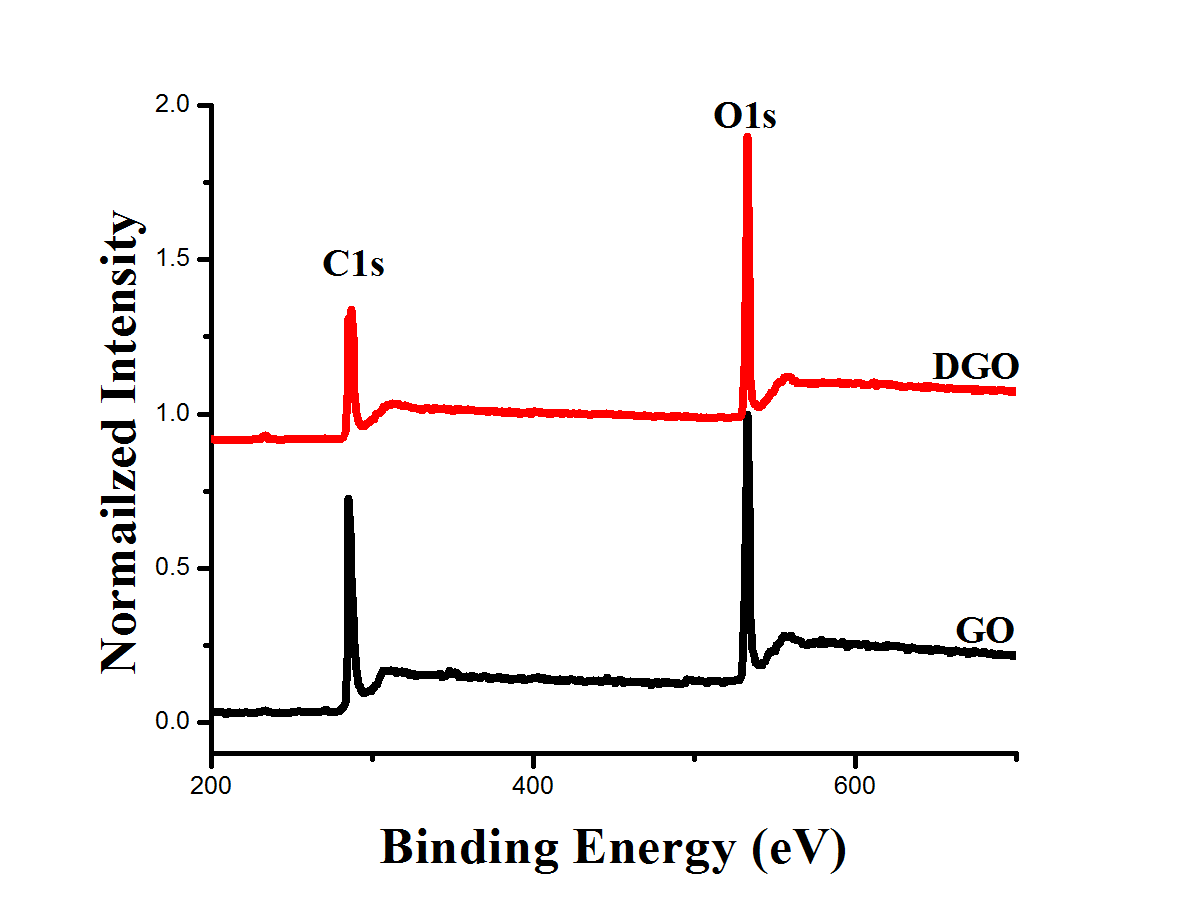


**Figure S2.** XPS survey spectra of DGO and GO.


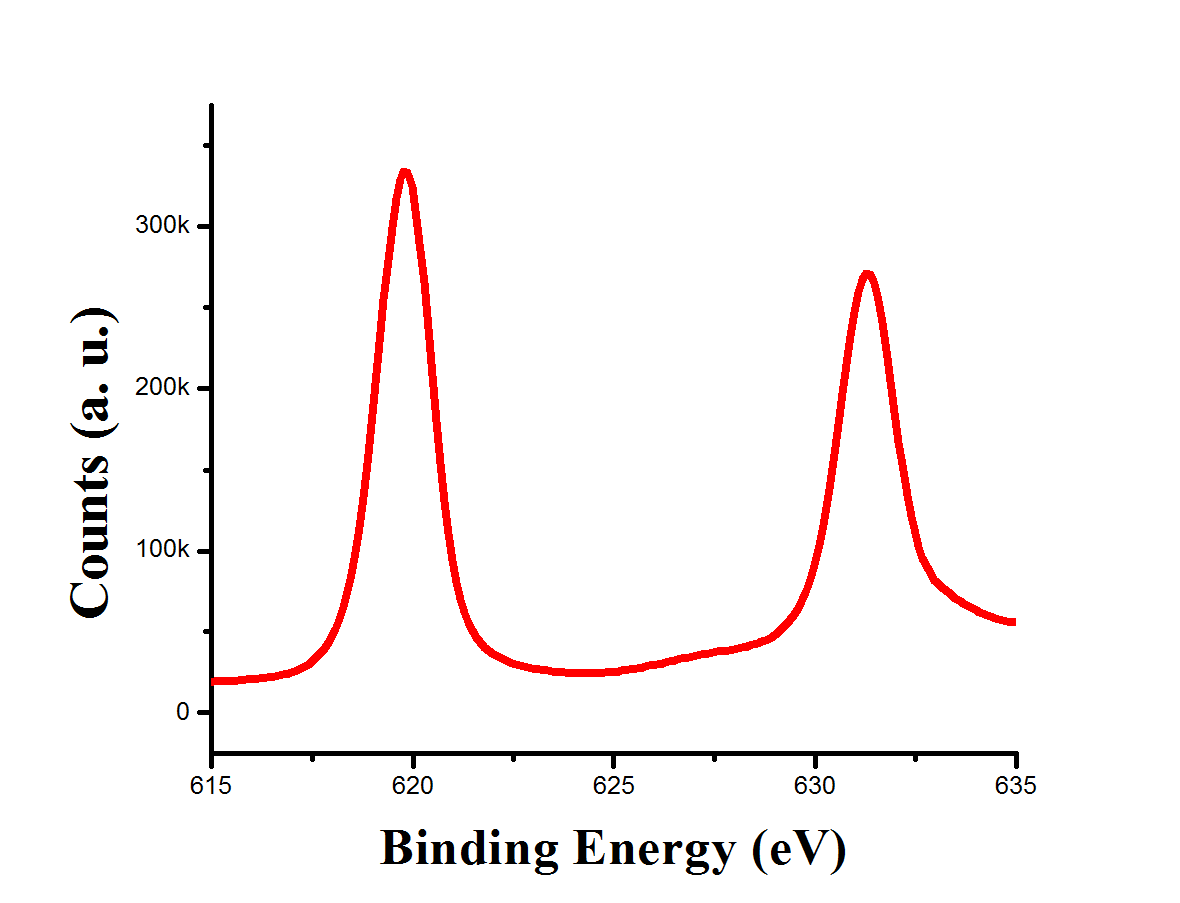


**Figure S3.** High-resolution I3d spectra of PVP-I.


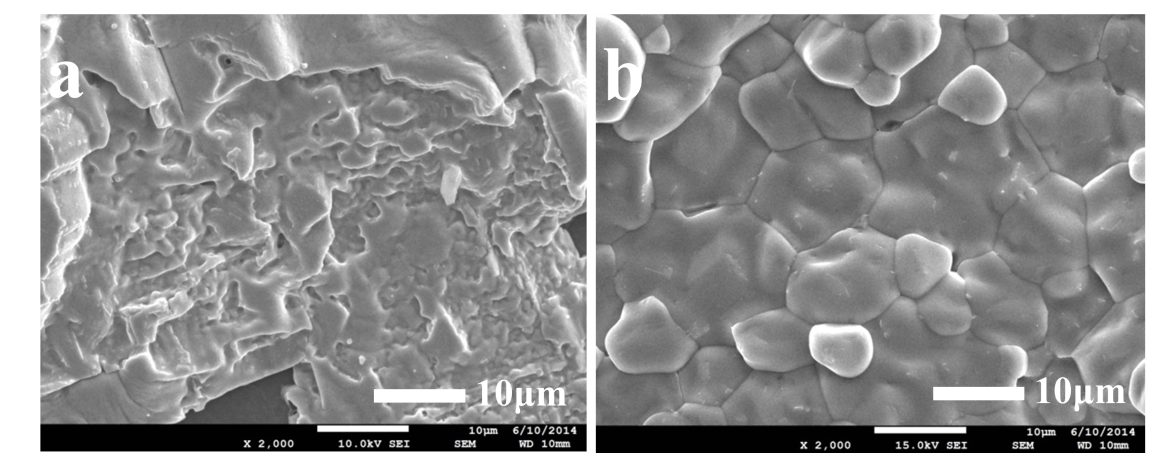


**Figure S4.** SEM images of a) PVP-I, and b) KI_3_.


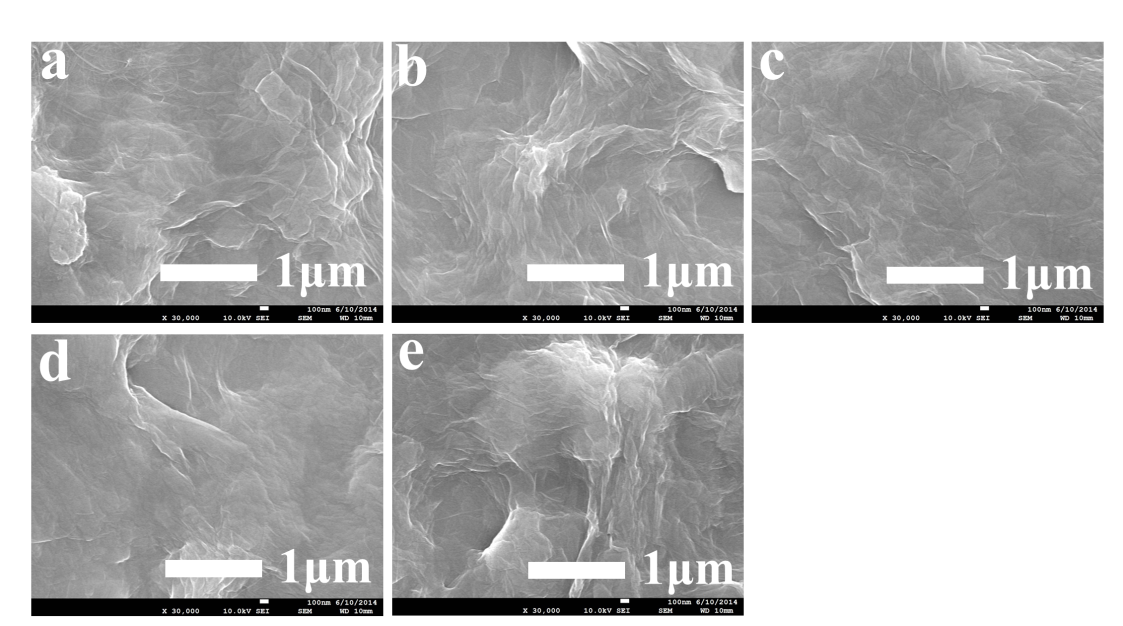


**Figure S5.** SEM images of DGO-I composite with no bacteria, Klebsiella pneumonia (a), Pseudomonas aeruginosa (b), Proteus mirobilis (c), Staphylococcus aureus (d), E. Coli (e) biofilms after 12 h.


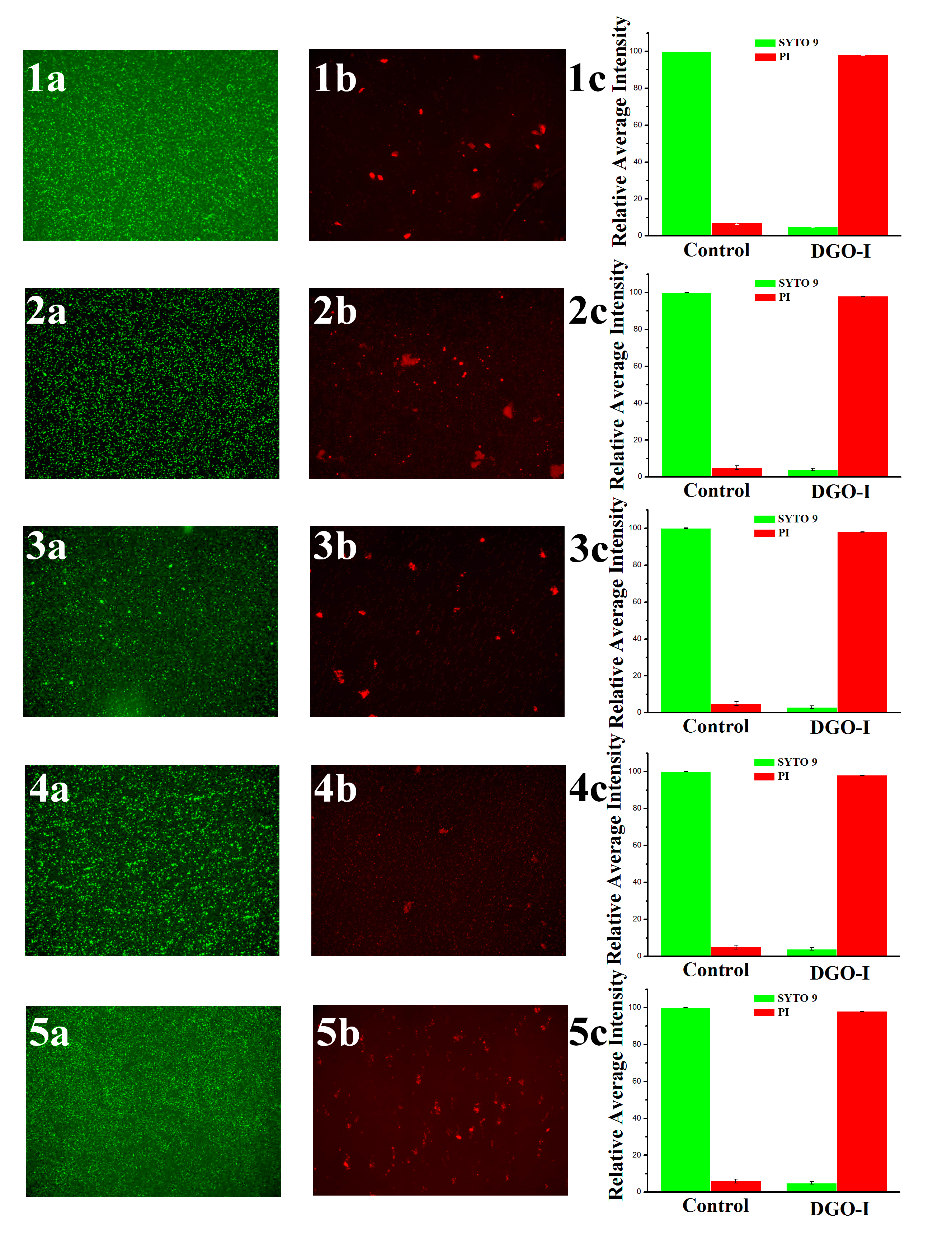


**Figure S6.** Live/dead assay fluorescent microscope images of E. Coli (1), Klebsiella pneumonia (2), Proteus mirobilis (3), Pseudomonas aeruginosa (4), and Staphylococcus aureus (5) (1a-5a, 1b-5b) . Images (1a-5a) for the control and (1b-5b) for the DGO-I-treated sample. Live cells fluoresce green and dead cells fluoresce red.Live/Dead assay quantification of the experiment shown in Figure 1c-5c.


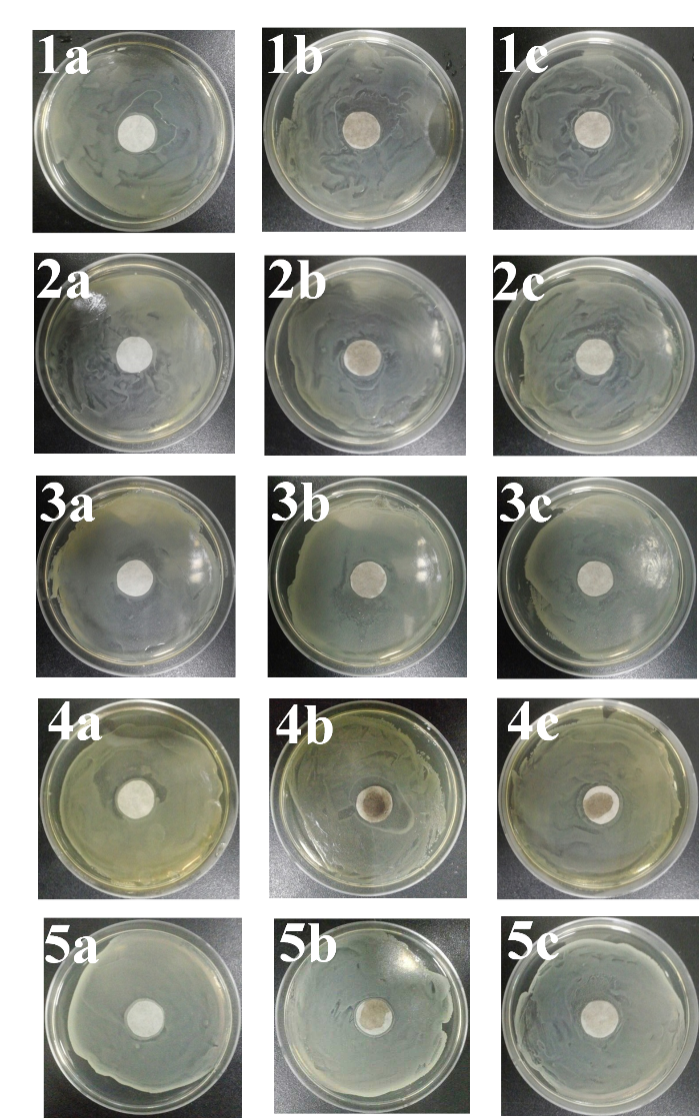


**Figure S7.** Photographs of the inhibition zone by the disk diffusion assay of E.coli (1), Klebsiella pneumonia (2), Pseudomonas aeruginosa (3), Proteus mirobilis (4), and Staphylococcus aureus (5) bacteria with (a) neat, (b) GO, and (c) DGO coated filter paper.


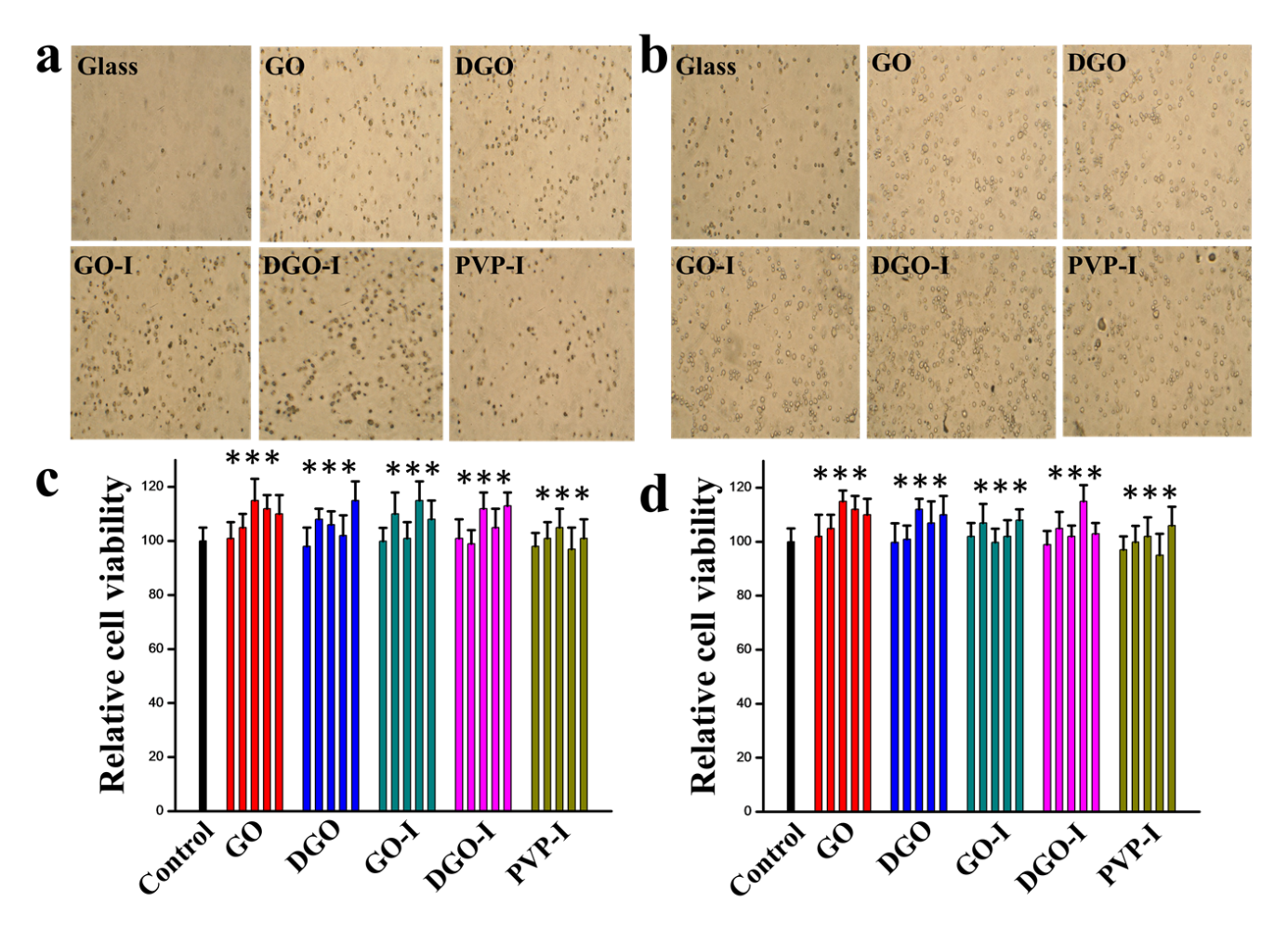


**Figure S8.** Growth of Human white hela (a) and MDA-MB-231 (b) cells on glass coverslips, GO, DGO, GO-I, DGO-I, and PVP-I (20×). *In vitro* concentration-dependent (100, 50, 25, 12.5, and 6.125 µg/mL, respectively from left to right) cell viability of HeLa (c) and MDA-MB-231 (d) cell lines. The cells were incubated with free GO, DGO, GO-I, DGO-I, and PVP-I for 24 h as indicated. Data represent mean ± Standard Error of the Mean (SEM)(n=6)(***p< 0.05 versus PBS, one-way ANOVA)

**Table S1.** Measurement of available iodine on filter papers with time.

|  | 1h | 2h | 3h | 4h | 5h | 6 | 7h | 8h | 9h | 10h |
| --- | --- | --- | --- | --- | --- | --- | --- | --- | --- | --- |
| GO-I (ppm) | 25 | 25 | 50 | 25 | 25 | 12.5 | 12.5 | <12.5 | 0 | 0 |
| DGO-I (ppm) | 50 | >50 | 50 | 50 | 50 | 25 | 12.5 | 12.5 | <12.5 | 0 |
| PVP-I (ppm) | 50 | 25 | 12.5 | 12.5 | 12.5 | 12.5 | <12.5 | 0 | 0 | 0 |

The sterile filter papers were dipped into GO-I, DGO-I, and PVP-I solution (1 mg/mL) and allowed to dry in a vacuum. The resulting filter papers were then placed on a sterile LB culture plate. Measurement of available iodine at the filter papers surface using iodine test paper containing starch and potassium iodide.
